# Supplementary material for: Value of low-keV virtual monoenergetic plus dual-energy computed tomographic imaging for detection of acute pulmonary embolism
Source: PLoS One. 2022 Nov 11;17(11):e0277060. doi: 10.1371/journal.pone.0277060 (PMC9651561; doi:10.1371/journal.pone.0277060)
Supplement: Attachment — Submitted filename: PLOS review.docx [file pone.0277060.s002.docx]

The manuscript does an excellent job of comparing between low energy VMI+ and standard dual energy CT for accurately diagnosing acute pulmonary embolism. It’s a well written and well represented manuscript. Even though the sample size is small (64 patient’s) authors did a great work in presenting their findings. Generalizability and validating the superiority of low energy VMI needs to be undertaken. Would advice authors to provide further information:

1) Any impact of BMI on diagnosing acute PE in the current sample
2) Advice authors to add BMI, h/o heart failure and possible sub group analysis for obese vs normal bmi group
3) For figure 1A and 1B please use arrow mark to point area of interest
